# Dr. Horace Babcock

**Published:** 1911-02

**Authors:** 


### OBITUARY

DR. HORACE BABCOCK, of Gowanda, N. Y., died at his home December 3, 1910, aged 86 years. He graduated at the Medical Department of the University of Buffalo in 1851. In 1862, then living in Wisconsin, he entered the military service of the United States as a medical officer of the second Wisconsin regiment of infantry, serving during the civil war. Afterward he located in Gowanda and there practised his profession until retiring a few years ago. His son, L. L. Babcock, is a prominent lawyer in Buffalo.

